# Protein *O*-GlcNAcylation in Cardiac Pathologies: Past, Present, Future

**DOI:** 10.3389/fendo.2018.00819

**Published:** 2019-01-15

**Authors:** Marine Ferron, Manon Denis, Antoine Persello, Raahulan Rathagirishnan, Benjamin Lauzier

**Affiliations:** ^1^Montreal Heart Institute, Montreal, QC, Canada; ^2^l'institut du thorax, INSERM, CNRS, UNIV Nantes, Nantes, France; ^3^Faculty of Health Sciences, University of Ottawa, Ottawa, ON, Canada

**Keywords:** *O*-GlcNAc, cardiovascular, ischemia-reperfusion, pharmacology, therapy

## Abstract

*O*-GlcNAcylation is a ubiquitous and reversible post-translational protein modification that has recently gained renewed interest due to the rapid development of analytical tools and new molecules designed to specifically increase the level of protein *O*-GlcNAcylation. The level of *O*-GlcNAc modification appears to have either deleterious or beneficial effects, depending on the context (exposure time, pathophysiological context). While high *O*-GlcNAcylation levels are mostly reported in chronic diseases, the increase in *O*-GlcNAc level in acute stresses such as during ischemia reperfusion or hemorrhagic shock is reported to be beneficial *in vitro, ex vivo*, or *in vivo*. In this context, an increase in *O*-GlcNAc levels could be a potential new cardioprotective therapy, but the ambivalent effects of protein *O*-GlcNAcylation augmentation remains as a key problem to be solved prior to their transfer to the clinic. The emergence of new analytical tools has opened new avenues to decipher the mechanisms underlying the beneficial effects associated with an *O*-GlcNAc level increase. A better understanding of the exact roles of *O*-GlcNAc on protein function, targeting or stability will help to develop more targeted approaches. The aim of this review is to discuss the mechanisms and potential beneficial impact of *O*-GlcNAc modulation, and its potential as a new clinical target in cardiology.

## Introduction

### Definition, Pathway, and Regulation

The *O*-N-acetyl glucosaminylation, commonly known as *O*-GlcNAcylation, is a reversible post-translational modification (PTM) that involves the addition of the monosaccharide β-D-N-acetylglucosamine to serine and threonine residues of proteins. It was first described by Torres and Hart on the internal surface of plasma membranes of lymphocytes (1). More recently, it has also been identified on cytosolic, nuclear, mitochondrial and membrane proteins (2–4). Over 3,000 proteins have been identified so far to be *O*-GlcNAcylated (5), and this number will probably increase with the development of new analytical techniques of *O*-GlcNAcylation detection such as the copper-catalyzed azide-alkyne cycloaddition “click” reaction described recently (6). From 1984 to 2004, <200 publications mentioning the MeSH terms “*O*-GlcNAc” or “*O*-GlcNAcylation” are referenced in Pubmed, while in 2017 alone almost 200 references can be found. Initially, little attention was paid to this minor sugar moiety, probably because the tools available to study this PTM were limited. In fact, detecting it is particularly difficult as it cannot be studied with classic techniques such as electrophoresis or high pressure liquid chromatography (HPLC) as the *O*-GlcNAc moiety has no impact on molecular weight or isoelectric point, and it is very labile (7). More recently, with the development of more specific pharmacological compounds, protein *O*-GlcNAcylation has regained attention. Whilst it is now evident that protein *O*-GlcNAcylation is involved in many pathologies (from cancer to neurological disorders and cardiac function), the overall impact of protein *O*-GlcNAcylation remains unclear as in some situations it is reported to be beneficial (e.g., ischemia/reperfusion) or deleterious (e.g., diabetes). This suggests a potential role of this PTM in adapting to stress response and its importance in pathophysiological situations.

**Figure 1:**
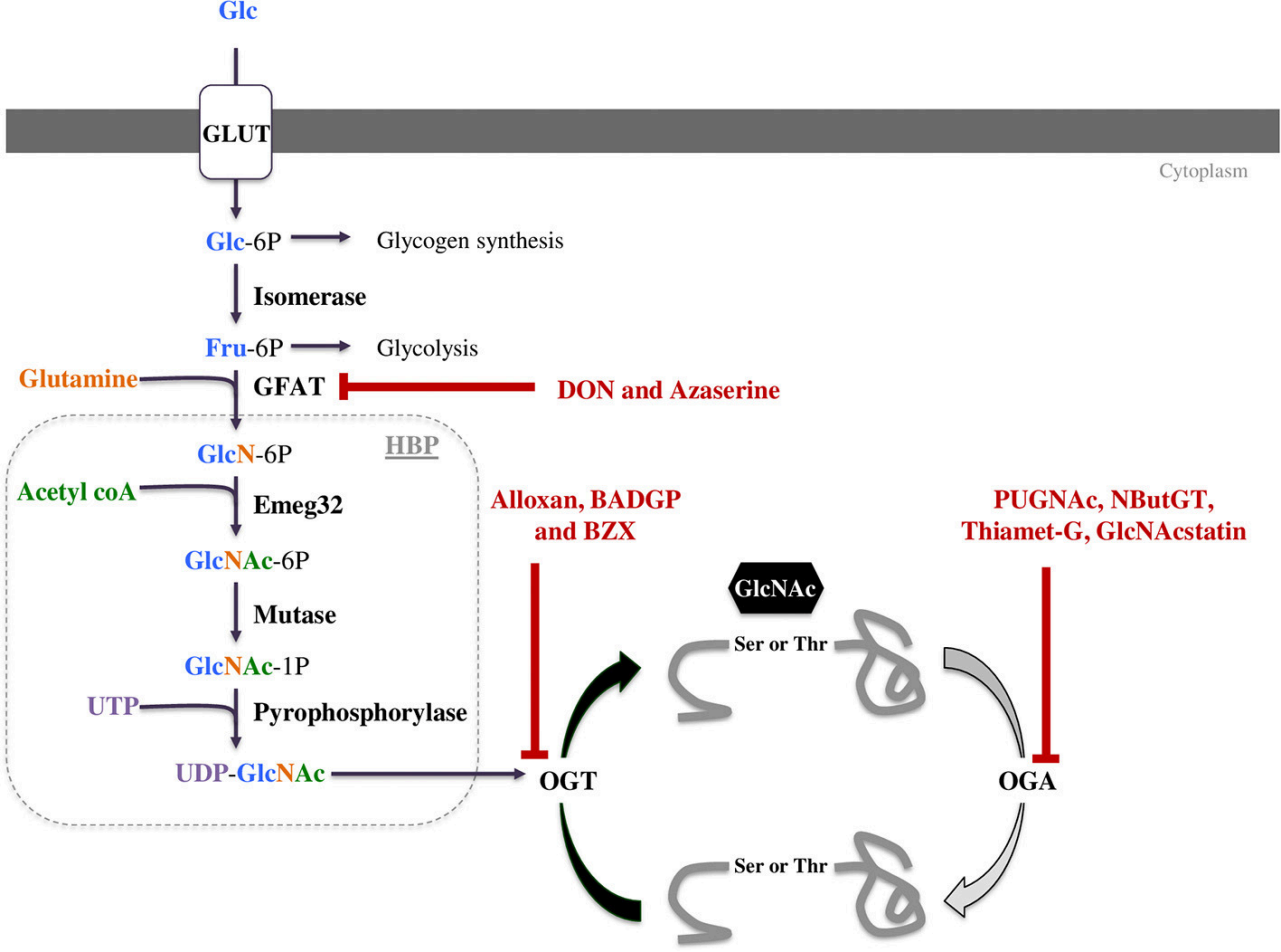
The hexosamine biosynthetic pathway (HBP) leads to UDP-GlcNAc formation and regulates *O*-GlcNAcylation. This pathway is regulated by only three enzymes: GFAT (glutamine fructose-6P aminotransferase), OGT (*O*-GlcNAc transferase) and OGA (*O*-GlcNAcase). These three enzymes can be targeted by pharmacological compounds to modulate *O*-GlcNAc levels. Some of them decrease *O*-GlcNAc levels such as DON and azaserine inhibiting GFAT or Alloxan, BZX and BADGP inhibiting OGT. OGA inhibitors like PUGNAc, NButGT, Thiamet-G and GlcNAcstatin increase protein *O*-GlcNAcylation.

Protein *O*-GlcNAcylation is regulated by the concerted actions of only three enzymes. When glucose enters a cell it can be metabolized in a number of different metabolic pathways including glycogen synthesis, the pentose phosphate pathway, the glycolysis, or the hexosamine biosynthetic pathway (HBP, Figure 1). The first enzyme, glutamine fructose-6P amidotransferase (GFAT) uses glutamine and 2 to 5% of glycolytic fructose-6-P to perform the first step of the HBP. As the rate-limiting enzyme, GFAT controls HBP flow and consequently the *O*-GlcNAcylation level (8, 9). Mammals express two GFAT isoforms, GFAT1 and GFAT2, which are coded by separate genes. Both isoforms are expressed in heart. GFAT1 is ubiquitous, and mainly expressed in placenta, pancreas, testis and skeletal muscle. GFAT2 shares 75% homology with GFAT1, and this isoform is mostly expressed in heart and the central nervous system. Once the UDP-GlcNAc group is formed, it can be added or removed from proteins by two enzymes: the *O*-GlcNAc transferase (OGT) and the β-*N*-acetylglucosaminidase (OGA), respectively (Figure 1). As for GFAT, different splice variants of OGT and OGA exist. Alternative splicing of *ogt* results in the generation of three isoforms, a nucleocytoplasmic (116 kDa-ncOGT), a smaller isoform (70 kDa) named short form (sOGT) and a mitochondrial (103 kDa-mOGT) isoform of OGT. It is unclear if this last isoform is active. The first two isoforms are expressed mainly in the cytoplasm and nucleus as heterotrimers consisting of 2 ncOGT and 1 sOGT subunits (8). Two isoforms of OGA have been formerly described, a long one (lOGA) of 102 kDa found in the nucleocytoplasm, and a shorter one (sOGA) of 76 kDa resulting from alternative splicing. The smaller isoform is found in the sarcoplasmic reticulum and lipid droplets and is less active (4). The existence of a functional mitochondrial OGA isoform is debatable and represents an important area of ongoing research.

### Pharmacological Regulation

The development of pharmacological tools to modulate protein *O*-GlcNAcylation has been very challenging. As discussed below, potent OGA inhibitors are now available for clinical use, but OGT inhibitors still need further development to be used properly *in vivo*. Altogether, these new molecules have allowed extensive characterization of this PTM.

#### Decrease in *O*-GlcNAc Levels

##### GFAT inhibitors

In the 1950's, when *O*-GlcNAc moiety was not yet discovered, *O*-diazoacetyl-L-serine (azaserine) and 6-diaz*O*-5-ox*O*-L-norleucine (DON) were developed to reduce tumor growth (10, 11). These molecules are structural analogs of glutamine and act as competing agonists or antagonists, respectively. They react with the catalytic region of most amidO-transferases, among them GFAT. They have a very low selectivity for GFAT. Whilst these compounds were used to efficiently induce antineoplastic effects, they presented a number of side effects including nausea and vomiting or loss of enthusiasm (12), and DON also causes hepatotoxicity in children (13). Moreover, through amid*O*-transferase inhibition, these compounds had pleiotropic effects in cells (Table 1). Developing new GFAT inhibitors represents a challenge because UDP-GlcNAc is also used for other cellular process, and its inhibition could have many side effects. As a result more attention has been paid to the development of OGT and OGA inhibitors.

**Table 1:** Summary of molecules currently available for *O*-GlcNAc level modulation: their actions, specificity, known limits and strengths.

**Effects**	**Action**	**Molecule**	**IC50**	**Strengths**	**Weaknesses**	**References**
*O*-GlcNAc decrease	GFAT inhibition	DON		Uses in oncologic research for its anti-neoplastic properties	Low selectivity for GFAT, toxic effects, pleiotropic effect	(10–14)
		Azaserine				
	OGT inhibition	Alloxan	18 μM	Cell permeant through glucose transporter (GLUT2 in pancreatic beta cells)	Off target effects, toxicity, induced ROS production, instable at physiologic pH (half-life 1,5 min)	(14–16)
		BZX	10 μM	Cell permeant, anti-cancer and anti-viral properties	Harmfull effects on cellular process	(17–20)
		BADGP			Abnormal *O*-glycosylation	(21–23)
		Ac-5SGlcNAc	5 μM	No modification of lectin glycosylation, uses in oncologic research	No use in *in vivo* conditions	(24, 25)
*O*-GlcNAc increase	UDP-GlcNAc increase	Glutamine		Used in hospital	Poor efficiency, pleiotropic effects	(9, 26–28)
		Glucosamine				
	OGA inhibition	PUGNAc		First OGA inhibitors synthetized	Poorly specific(OGA/HEX = 1), desensitizes cell to insulin, doesn't cross blood brain barrier	(14, 29)
		NButGT	8 μM	High specificity(OGA/HEX = 1,500)	Lack of stability *in vivo* (half clearance 30 min), limited chemical stability in solution	(30, 31)
		Thiamet G	30 nM	High specificity(OGA/HEX = 35,000)	Expensive	(31)
		GlcNAcstatin G	4 nM	High specificity(OGA/HEX = 900,000)	Expensive, lack of study	(32, 33)

*OGA, O-GlcNAcase; OGT, O-GlcNAc transferase; GFAT, glutamine fructose-6P amidotransferase; DON, 6-diazO-5-oxO-L-norleucine; BADGP, benzyl-2-acetymidO-2-deoxy-α-D-galactopyranoside; BZX, benzoxazolinones; IC 50, concentration of inhibitor required for achieving 50% inhibition; GLUT 2, Glucose Transporter Type 2*.

##### OGT inhibitors

Different types of OGT inhibitors exist (Figure 1, Table 1). In this review, we will only focus on the four most described and used molecules. (1) Alloxan is a uracil analog developed at the beginning of the Twentieth Century (34). It was shown to be an OGT inhibitor on isolated pancreatic islets *in vitro* (35), and is absorbed via the glucose transporter GLUT2 in pancreatic β-cells. However, alloxan is highly unstable at physiological pH (half-life 1.5 min), it drives cell toxicity due to ROS production, and is not OGT specific as it also inhibits OGA (14–16). (2) At the end of the 1960's, benzoxazolinones (4-methoxyphenyl 6-acetyl-2-ox*O*-2,3-dihydr*O*-1,3-benzoxazole-3-carboxylate, or BZX) and their derived-compounds were proposed as potential new therapeutics due to their anti-cancer and anti-viral properties (17–19). However, as BZX irreversibly inactivates OGT, it cannot be used clinically due to its potential harmful effects on other cellular process (20). (3) BADGP (benzyl-2-acetymid*O*-2-deoxy-α-D-galactopyranoside), a N-acetylgalactosamine derivative, was also used as an OGT inhibitor (36, 37). Permanent exposure to BADGP induced abnormal *O*-glycosylation of mucins in HT-29 cells with potential negative effects on host defenses against pathogens (21–23). (4) Finally, a last inhibitor: Ac-5SGlcNAc, was proposed in 2011. This compound produced 5S-UDP-GlcNAc that binds to OGT and inhibits its activity. Authors demonstrated dose and time-dependent effects of this inhibitor (from 0.1 to 1,000 μM) and an EC50 at 5 μM in COS7 cells. These results were confirmed in fibroblasts, hepatocytes and neuronal cells, and by others in different cell types especially in an oncologic context. Contrary to the three first OGT inhibitors, Ac-5SGlcNAc does not perturb lectin glycosylation even at the highest dose. Thus, this compound is more frequently used in basic research, however, there is, today, no evaluation of its *in vivo* efficiency and safety (24).

In summary, whilst OGT inhibitors currently lack specificity and selectivity, improvements in OGT inhibitors is necessary to be able to reduce protein *O*-GlcNAc levels and represents a potentially huge opportunity to reduce cancer or diabetes burden.

#### Increase in *O*-GlcNAc Levels

The development of strategies to increase *O*-GlcNAc levels has been more successful and can be used in human. Two different approaches are available to increase protein *O*-GlcNAc levels: increase in total UDP-GlcNAc through an increase in HBP flux or pharmacological inhibition of OGA.

##### Increase in UDP-GlcNAc concentration

Glucosamine (GlcN) bypass the rate limiting enzyme of the HBP, GFAT (38) resulting in higher UDP-GlcNAc levels, consequently it increases protein *O*-GlcNAcylation. Yet, if GlcN increases protein *O*-GlcNAc levels by 2 or 3-fold, this compound also has side effects such as: decrease in ATP production or increase in proteoglycans production (39, 40).

##### OGA inhibitors

Another way to increase protein *O*-GlcNAcylation is to inhibit OGA, and different molecules have been developed over the last 20 years. (1) The first described in the literature is the *O*-(2-acetamid*O*-2-deoxy-D-glucopyranosyliden)-amin*O*-N-phenylcarbamate commonly known as PUGNAc. It has been the most widely used compound to inhibit OGA for about a decade. However, PUGNAc also reacts with other hexosaminidases (HEX) such as lysosomal β-hexosaminidases with an inhibition ratio OGA/HEX of 1 (29). Recent OGA crystallography studies lead to the development of specific OGA inhibitors (31). These molecules interact directly with the active site of OGA, and include NButGT, Thiamet-G, and GlcNAcstatins. (2) NButGT (1,2- dideoxy-2'-propyl-α-D-glucopyranos*O*-(2,1-d)-Δ2'-thiazoline) is a competitive inhibitor of OGA and has good efficiency and specificity (Table 1). However, according to a study by Macauley et al. NButGT has a half-clearance of only 30 min *in vivo*, and lacks stability in solution (few days to weeks) (30). (3) Thiamet-G [(3aR,5R,6S,7R,7aR)-2-ethylamin*O*-3a,6,7,7a-tetrahydr*O*-5-(hydroxymethyl)-5H-pyrano(3,2-d)thiazole-6,7-diol] was developed several years later, and is more stable (31). (4) More recently, GlcNAcstatin has been described. It presents a molecular architecture noticeably similar to PUGNAc (Table 1) (32, 33), with a high selectivity and efficiency. Unfortunately, synthesis of GlcNAcstatin remains complex and very expensive (41).

Over the last decade, pharmacological modulators of *O*-GlcNAc levels have been developed for (i) improved knowledge of physiological conditions, as well as (ii) potential utilization as therapeutic strategies in different pathologies.

## Potential Impact of Modulating *O*-GlcNAc Levels in Pathologies

The consequence of an increase in protein *O*-GlcNAcylation has mainly been evaluated in diabetes or cancers. In these pathologies, patients with high *O*-GlcNAc levels present with the poorest outcome. In this context, a reduction in *O*-GlcNAc levels appears to be an interesting therapeutic strategy. Alternatively, in acute pathologies, *O*-GlcNAc stimulation using different approaches to increase *O*-GlcNAc levels could be a promising therapeutic approach.

### Increasing *O*-GlcNAc Levels in Acute Pathology, a Potent Therapeutic Approach?

Several studies have demonstrated the importance of *O*-GlcNAc response to a stress, and especially an increase in *O*-GlcNAc levels following this stress. This augmentation is reported to improve cell survival through a decrease in pro-apoptotic pathway molecules (p53, FOXO3, caspase 8 or GPAT1) and activation of sirtuin deacetylase (SIRT1) (42–45). Similarly, the beneficial effects of protein *O*-GlcNAcylation stimulation, with GlcN or siOGA, are associated with a decrease in apoptosis (46–48). These *in vitro* results were confirmed *in vivo* for different types of stress (e.g., hypoxia, inflammation, oxidative stress), and in different tissues and/or different pathologies.

In kidney, damage caused by hypoxia or an acute injury using a rabbit model are attenuated by GlcN administration (49). Hu et al. also reported an improvement in renal function and a decrease in apoptosis and oxidative stress markers, and these effects were abolished with alloxan (45). In a brain model, using a middle cerebral artery occlusion (MCAO), it was shown that an increase in *O*-GlcNAc levels, by GlcN or Thiamet G, resulted in reduced infarct volume and an improvement in cognitive function. These effects could be explained by a reduction in apoptosis and inflammation (suglia and NF-κB activation, reduction of cytokine production and leukocyte infiltration) (50–52). In hemorrhagic shock, hypovolemia is associated with an alteration in glucose utilization. Using *in vitro* (neonatal rat ventricular cardiomyocytes) and *in vivo* (rat) models of hemorrhagic shock Chatham's group demonstrated that augmentation of *O*-GlcNAc levels improved global outcomes. Specifically, GlcN improved organ perfusion, cardiac function, the inflammatory state and finally, increased survival (53, 54). Several years later, these results were confirmed with a specific inhibitor of OGA, PUGNAc, validating the potential therapeutic role of protein *O*-GlcNAcylation in this pathology (53, 55). In all these studies, cardiovascular function was significantly improved.

### Ischemia-Reperfusion From *ex vivo* to *in vivo*

Myocardial infarction results from an obstruction of a coronary artery creating an ischemia leading to tissue necrosis. Reperfusion, by thrombolysis or invasive procedures, is the only way to preserve cardiac function and to save patient life and must be performed as early as possible and within the first 12 h. Unfortunately, reperfusion exacerbates cardiac injury through an excessive oxidative stress and inflammatory response. In this context, the introduction of an infarct-limiting therapy in clinical practice might have a clinical and socioeconomic impact (56).

The first evidence of the potential beneficial effects of *O*-GlcNAc was shown *in vitro*. Champattanachai and collaborators reported that an increase in *O*-GlcNAcylated proteins through GlcN infusion in rat neonatal cardiomyocytes improved cell viability following IR. Moreover, with the use of different pharmacological compounds (glucosamine, PUGNAc, azaserine, alloxan), they demonstrated a positive correlation between *O*-GlcNAc levels and cell viability in IR. According to the authors, the beneficial effects of GlcN are associated with a decrease in calcium overload and apoptosis through a reduction in mitochondrial permeability, transitional pores, or mPTP opening (57, 58). A second team confirmed these results by specifically targeting OGA. They modulated *O*-GlcNAc levels by PUGNAc, adenoviral overexpression of OGA or siRNA's against OGA, and demonstrated that an increase in *O*-GlcNAc levels increased cell viability and decreased apoptosis and oxidative stress in responses to IR (59, 60).

To confirm these initial *in vitro* findings, *ex vivo* studies have also been performed. A first study using a Langendorff model of IR demonstrated that glutamine improved cardiac function through an improvement of left cardiac function (ventricular pressures and heart rate) and a decrease in infarct size (cardiac troponin I release). According to these authors, this could be explained by a restoration in cellular ATP concentration (61). Once again, these beneficial effects were confirmed using an OGA inhibitor, NAG thiazolines, and this compound also reduced infarct size and mechanical arrhythmic activity (62).

Intriguingly, the number of *in vivo* studies is quite limited and only focuses on murine models. Considering the potential clinical impact of *O*-GlcNAc stimulation on cardiac function, it remains as an important step to continue toward clinical validation. The only direct *in vivo* evidence confirming *in vitro* results is from mice treated with PUGNAc at the reperfusion stage. This treatment efficiently reduced infarct size, apoptosis and mPTP opening (63). However, many *in vivo* studies indirectly suggest that *O*-GlcNAc stimulation could improve patient outcomes. For example, hearts submitted to preconditioning (two periods of 5 min ischemia and 5 min reperfusion) presented a higher myocardial glucose uptake and a higher protein *O*-GlcNAcylation, and a better recovery. In this context, the authors explained that the *O*-GlcNAc level increase was responsible for the cardioprotective preconditioning effect (64).

## TOWARD a Potential Clinical Application

### From Bench to Bedside, a Complicated Step

Several studies in cellular and animal models have demonstrated the potential beneficial effects of *O*-GlcNAc level augmentation in acute pathologies, and especially in cardiac IR. However, several limitations still exist and these need to be studied before a potential clinical application. Whilst acute *O*-GlcNAc level augmentation is cardioprotective in murine models, the adverse effects of a long-term exposure to high *O*-GlcNAc levels should also be considered. For instance, hearts isolated 1 month after myocardial infarction induced by coronary ligation presented high levels of protein *O*-GlcNAcylation, and especially higher *O*-GlcNAcylation of troponin T, and this observation was linked to higher cardiac dysfunction (65). Similarly, in rats subjected to hypoxic conditions (alternating 2 min 21% O_2_ and 2 min 6–8% O_2_ 8 h per day) *O*-GlcNAc levels started to rise 2 weeks after the first stress. This observation was associated with higher apoptosis and inflammatory markers (66). Myocardial infarction and the resulting reperfusion injury is associated with cardiac remodeling, hypertrophy and heart failure, a situation aggravated by high *O*-GlcNAc levels, even if there is still no consensus regarding the link between *O*-GlcNAcylation of proteins and cardiac hypertrophy (67, 68). However, several studies have demonstrated an increase in cardiac protein *O*-GlcNAcylation in *in vitro* and *in vivo* models of hypertrophy (69–73). *O*-GlcNAc levels in the left ventricular myocardium were increased in patients with heart failure (73). Furthermore, increases in *O*-GlcNAc levels were associated with heart failure development (65, 74). In parallel, augmentation of protein *O*-GlcNAcylation turns out to be an adverse therapy for diabetic patients. In diabetic IR conditions, hyperglycemia and high *O*-GlcNAc levels are also associated with an aggravation of cardiac dysfunction and infarct size (75, 76).

In summary, before clinical trials can be conducted, more studies are necessary to characterize the potential long-term impact of *O*-GlcNAc stimulation and to evaluate the best dose, time-point and duration of treatment to avoid adverse effects.

### To Future Potential Clinical Trial in Cardiology

Glutamine and glucosamine are metabolites used in the HBP pathway and could be a way to increase *O*-GlcNAc levels via an increase in HBP flux. These two molecules are already used clinically to treat inflammatory disease or improve cardiac function, and could provide proof of potential utilization in cardiac acute pathologies like IR. Despite this, their impact on *O*-GlcNAc level has never been tested in humans.

Glutamine supplementation is now recommended for parenteral or enteral supplementation in neonatal, pediatric and adult intensive care units and seems to be safe (26, 27). Moreover, in pediatric or adult intensive care units, patients with low plasma glutamine (<420 μmol/l) at admission are at higher risk of mortality and increased incidence of multiple organ failure (77, 78). Whilst this observation suggests a potential benefit from increasing glutamine concentration the consequence on protein *O*-GlcNAcylation has never been studied. Enteral glutamine supplementation has been shown to reduce the incidence of serious neonatal infections in preterm and/or very low birth weight children (79) and enterocolitis (80, 81). In critical conditions, glutamine enteral supplementation decreases the incidence of sepsis, pneumonia, and bacteremia in trauma (82) and burn patients (83). Moreover, glutamine may have a perioperative cardioprotective role. Glutamine use in patients with ischemic heart disease operated under conditions of extracorporeal blood circulation or cardiopulmonary bypass reduces troponin release at day 1, the systemic vascular resistance index and improves cardiac and stroke index (84, 85). As well, perioperative glutamine supplementation during aortic surgery can compensate renal arginine synthesis loss induced by aortic clamping and could also improve post-operative renal function (86). A recent clinical trial showed that patients who receive oral glutamine have less complications, myocardial damage, morbidity and mortality after coronary revascularization under cardiopulmonary bypass (87). A similar protocol in chronic angina patients, delayed the time to onset of more than 1.0 mm of ST segment depression on the electrocardiogram (ECG) by 38 s, but did not improve hemodynamic response to exercise, the time of onset of angina symptoms, maximum workload or total exercise time (88).

GlcN is largely used for osteoarthritis at an average dose of 1,500 mg per day but its usefulness in other pathologies has not been explored in clinical trials. Whereas, long term treatment seems to delay the progression of knee arthritis (28), multiple studies have shown no superiority of glucosamine vs. placebo (89), no improvement of cartilage damage (90) and no role in prevention of osteoarthritis in overweight women (91). Moreover, at the usual doses, GlcN may induce an increase in intraocular pressure (92). This possible deleterious effect of high dose GlcN is supported by a recent review that showed this molecule is not beneficial for all population subgroups (93). Clinical use of GlcN has been associated with potential side effects *in vivo*, among them vomiting and diarrhea (94) and has also been associated with intracellular ATP depletion (95). Overall, GlcN utilization for joint pain appears to be safe.

Glutamine or GlcN supplementation has demonstrated its benefits for a few pathologies but these molecules are not specific to the HBP, and the link between the benefits and an increase in protein *O*-GlcNAcylation has not been demonstrated. Recently, new molecules targeting OGA have been studying for the treatment of tauopathy. MK-8719, a selective and potent small molecule inhibitor of OGA has shown promising results in the treatment of tauopathy such as Progressive Supranuclear Palsy (PSP). It has been evaluated in a recent phase I study in healthy volunteers. Interestingly, MK-8719 administration elicited PBMC *O*-GlcNAcylated protein increases in a dose dependent-manner, consistent with preclinical observations. Moreover, ASN120290, a brain-permeable small-molecule OGA inhibitor, has been also studied in a randomized, double-blind, placebo-controlled phase I study. These molecules seem to be safe and well tolerated (96).

These molecules represent a huge opportunity for progression to future clinical trials. Despite the beneficial impact of an increase in total *O*-GlcNAc level in IR, the treatment remains non-selective and can have potential side effects that have not been clearly evaluated in most studies. In future studies, more attention should be paid to doses and administration time to avoid any drawback.

### What Should be Confirmed Prior to Performing Clinical Trials?

#### Understanding Metabolism and *O*-GlcNAc Level Variation Through Aging

*O*-GlcNAc levels are linked to GFAT activity and cellular metabolism, particularly that of glucose. This observation is particularly important in cardiac tissue as cardiac metabolism constantly adapts to conditions and evolves throughout the first stage of life, especially the substrate selection for energy production. The predominant substrates for energy production in fetal hearts are carbohydrates (mainly glucose, lactate and pyruvate) and cardiac metabolism is mainly anaerobic. After birth, the ability of hearts to oxidize fatty acids increases within the first week (97), and in the adult heart, energy is mainly supplied by fatty acid oxidation (60–80%), carbohydrates (20–30%) and ketone bodies (10%). These proportions are constantly modulated to fit requirements and substrate availability. During fetal life, cardiac glucose uptake is controlled by a low affinity insulin-independent glucose membrane transporter, Glucose transporter type I (GLUT1). Shortly after birth, cardiac glucose transporters switch from the GLUT1 isoform to the GLUT4 isoform. GLUT4 is an insulin-sensitive glucose transporter, which is the predominant transporter in the adult heart (97). This observation is of particular importance as changes in glucose transporter expression, such as GLUT1 and GLUT4, have been shown to influence UDP-GlcNAc levels in mice. Interestingly, GFAT activity and glucose flux via the HBP are increased in muscles of GLUT1-overexpressing mice but not GLUT4-overexpressing mice (98). Consequently, during the first days of life, cardiac metabolism is subjected to dramatic changes with a major increase in fatty acid oxidation and a reduction in carbohydrate metabolism, whilst the impact on protein *O*-GlcNAcylation remains unknown. Altogether, the metabolic modification associated with the first days of life could impact *O*-GlcNAc levels and be of great importance in cardiac development and maturation. They could also be responsible for the higher capacity of the heart to withstand stress during the first days of life (99–101). Furthermore, in situations of stress or specific pathological conditions, the proportion of carbohydrates metabolized will increase to sustain cardiac needs. In the long run, this modification will affect HBP flux and modulate *O*-GlcNAc levels resulting in a potential impact on cardiac function. Interestingly, no study has evaluated if these modifications actually have an impact on cardiac *O*-GlcNAcylation.

While metabolism is subject to modifications throughout aging, and while protein *O*-GlcNAcylation has been described as a metabolic sensor, the exact link between metabolism's age-associated variations and *O*-GlcNAc levels has never been explored. In fact, many authors have only focused on senescence, and demonstrated that *O*-GlcNAc levels have an impact on the development and the progression of chronic diseases (102, 103).

**Figure 2:**
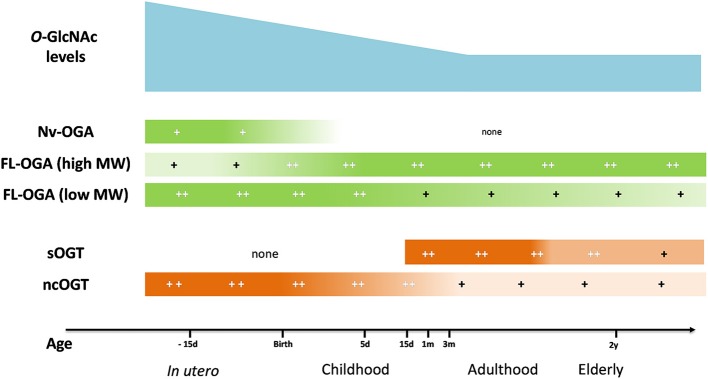
Evolution of *O*-GlcNAc levels, OGA and OGT expression in brain tissue throughout lifespan. OGA, *O*-GlcNAcase; OGT, *O*-GlcNAc transferase; FL-OGA, full-length OGA; high MW, high molecular weight, low MW, low molecular weight; Nv-OGA, Nuclear variant OGA; ncOGT, nucleo-cytoplasmic OGT; sOGT, short OGT;−15 d, 15 days before birth; 5 d, 5 days post-natal; 15 d, 15 days; 1 m, 1 month; 3 m, 3 months; 2 y, 2 years (105–107).

The variation of *O*-GlcNAc levels during aging has not reached a consensus yet. Fülöp et al. showed a decrease in *O*-GlcNAc levels in rat hearts which was associated with a reduction in OGT expression between adolescence (6 weeks) and adulthood (22 weeks) (104). However, in another study, the authors showed an increase in *O*-GlcNAc levels in rat hearts between 5 and 24 months, which surprisingly, was associated with a decrease in OGT expression (105). In the brain, which is the most studied organ for this question, age-associated variation of protein *O*-GlcNAcylation is not clear. *O*-GlcNAc levels rapidly decrease between the 1st and the 24th month (106) or increase between 5 and 24th month (105), whereas Rex-Mathes et al. showed no change in protein *O*-GlcNAcylation between the 3rd and 13th month (107) (Figure 2).

The lack of consensus on *O*-GlcNAc level variation throughout aging and in organ function underlines the need for new standardized studies to improve the understanding of *O*-GlcNAc levels on cellular development, survival and potential impacts on pathology or treatment responses.

#### Doses and Timing of Treatment

The ambiguous effects of *O*-GlcNAc is not restricted to the differences in basal *O*-GlcNAc levels, as it is now evident that there are dose-dependent effects of protein *O*-GlcNAcylation. The beneficial effects associated with increase in *O*-GlcNAc are lost for high increase in *O*-GlcNAc level. Gu et al. showed for brain IR that a high increase in *O*-GlcNAc levels (~7 fold) compared to a moderate increase in *O*-GlcNAc level (~3 fold) leads to a detrimental effect with an increase in infarct size. Strikingly, most of the beneficial effects described in the literature are associated with a 2–5 fold increase in protein O-GlcNAcylation levels in the heart (45, 57, 61), and in the brain (51, 108–110). Champattanachai et al. showed similar results, and highlighted a close link between O-GlcNAc levels and cell viability in their model (57). Unfortunately, few studies have focused on O-GlcNAc level modulation in physiological or pathophysiological conditions. In addition, the same authors showed that GlcN increases the level of protein *O*-GlcNAcylation by 1.5 fold in normoxia, and by 3 fold in hypoxia (57). The stimulation of this PTM could induce different responses in pathophysiological conditions. Interestingly, a recent clinical study showed that delayed sepsis treatment using 0.35 g/kg of glutamine per day *i.v*. and 30 g per day glutamine given via enteral administration to patients with two or more failing organs, had no effect on the outcome of organ failure and infections. It even increased mortality in hospital and at 6 months (111). These results might be explained by the high dose of glutamine (dose-dependent effect) and/or the late initiation of the protocol (time-dependent effect).

The lack of data on dose and time of treatment remains the key safety issue that needs to be satisfied in order to progress toward a clinical trial. Only one study has focused on establishing an optimal glutamine supplementation dose in pediatric cancer patients, however they did not evaluate the impact on *O*-GlcNAc levels (112).

## Improvement of Tools

In order to improve the understanding of the impact of *O*-GlcNAc on proteins and cell survival considerable progress has been made in developing new tools. Three main themes are of particular importance and have gained momentum over the last couple of years: (i) identifying *O*-GlcNAcylation sites on protein using mass spectrometry, (ii) improving pharmacological tools to improve specificity and biocompatibility, and (iii) understanding the impact of *O*-GlcNAcylation on specific proteins.

### Emerging Analytical Tools

As discussed above, there is a significant conundrum for protein *O*-GlcNAcylation, with on one-hand, potential short-term beneficial effects and on the other hand long-term deleterious effects. It is important to decipher which *O*-GlcNAc sites are of interest and potentially beneficial or potentially detrimental. Hopefully, in the near future, specifically targeting these sites will potentiate the beneficial effects and limit adverse effects associated with untargeted increases in *O*-GlcNAc level. Global mapping of *O*-GlcNAcylated proteins and peptides has recently been possible using mass spectrometry approaches. Recent advances in analytical techniques have also allowed to determine *O*-GlcNAcylation sites. For example, Thompson et al. highlighted “emerging technologies for quantitative, site-specific MS-based *O*-GlcNAc proteomics (*O*-GlcNAcomics), which allow proteome-wide tracking of *O*-GlcNAcylation dynamics at individual sites.” In this article, the authors listed the current technique for *O*-GlcNAc identification using mass spectrometry (6). Many papers have described the successful use of a combination of fractionation and click chemistry to label and identify *O*-GlcNAc sites on proteins. For example, Griffin et al. extensively studied the *O*-GlcNAcylation site on OGT with a chemically cleavable tag and suggested a potential implication of *O*-GlcNAc on the regulation of protein function (113). More recently, Deracinois et al. used this approach on skeletal muscle proteins and reported that some *O*-GlcNAcylation sites were located in interaction sites that open new area of research for this PTM (114). Thanks to the development and thorough validation of these new tools, consensus sequences have recently been proposed (115). Recent advances in technologies represent a huge opportunity and will definitely help to improve the understanding of the role of this PTM.

### Toward Specific Pharmacological Tools

Pharmacology has long been used to study different pathways and targets, and OGA, GFAT and OGT inhibitors have been known about since the early 90's. At present however, the main drawback of these compounds are their lack of specificity and affinity and their toxicity.

OGA inhibitors have been most beneficial and are now available for clinical trials. In fact, they have been the prime targets to develop a therapeutic strategy (29). The remaining challenge is in the potential directed system administration, in order to limit the potential off target effects. While the use of enzymatically triggered prodrugs is well known in the field of cancer, this strategy is poorly developed for treatment of other pathologies. In parallel, a second strategy could be to design new OGA inhibitors targeting specific organelles (e.g., mitochondria) or organs. Such tools would present two major advantages as they would help to: (i) decipher the role of this PTM on different organelles and (ii) improve treatment specificity. On the other hand, while OGT inhibitors potentially represent an interesting therapeutic strategy for cancer, they still lack specificity, are not cell permeant or are toxic.

Recent advances in crystallography opens potentially new avenues to study the impact of *O*-GlcNAc levels on cellular function. The next step will be to specifically target cellular compartments and/or specific proteins to be able to maximize potential beneficial impacts on selected pathways or cellular processes (61, 115–118).

### Site Specific Evaluation

Recent advances in *O*-GlcNAc proteomics has produced a very large quantity of potential sites to explore. It is now important to develop screening tools to be able to further advance our understanding of protein *O*-GlcNAcylation. Because only two enzymes are involved in *O*-GlcNAcylation, a solution may rely on biochemistry, and more specifically on site-specific mutagenesis through the incorporation of non-natural amino acids (NAA) such as selenocysteine derivatives. NAA are useful tools to add new properties to proteins at specific positions. They can be incorporated into a protein sequence during translation through genetic code expansion by an orthogonal (i.e., not interfering with the natural amino acids system) aminoacyl-tRNA synthetase (aaRS). This TAG codon is positioned in an appropriate position into the recombinant proteins gene (119, 120). Protein function could then be evaluated through enzymatic assays for example.

## Conclusion

Over the last 30 years, knowledge on protein *O*-GlcNAcylation has increased considerably in many areas, yet, the cardiovascular field remains largely underexplored. A pubmed search using the “cardiovascular” and “*O*-GlcNAc” MeSH term retrieved only 187 papers in October 2018. More effort has been expended on chronic pathologies such as diabetes, cancer, and Alzheimers disease, leading to potential new approaches for the patient. On the acute side, augmentation of *O*-GlcNAc levels may represent a new therapeutic solution for cardiovascular dysfunction or ischemia/reperfusion, yet its potential harmful effects at higher doses, or the impact of long term stimulation remain to be determined. Furthermore, deciphering which protein or pathway is involved in *O*-GlcNAc effects represents the key element to be able to specifically target them. The future may be hidden in organelle specific *O*-GlcNAc modulation or in new proteomic approaches with powerful tools to study *O*-GlcNAcylation. Increasing the understanding of this very specific PTM will open complete new area of research for protein-targeted mutation.

## Author Contributions

MF, MD, AP, and RR wrote the review. BL corrected the final manuscript.

### Conflict of Interest Statement

The authors declare that the research was conducted in the absence of any commercial or financial relationships that could be construed as a potential conflict of interest.
